# Esophageal perforation after transesophageal echocardiography: A case report

**DOI:** 10.1016/j.ijscr.2019.11.020

**Published:** 2019-11-19

**Authors:** Facundo Iriarte, German Adriel Riquelme, Pablo Sorensen, Daniel Enrique Pirchi, Matias Mihura Irribarra

**Affiliations:** a General Surgery Department, Hospital Británico de Buenos Aires, Perdriel 74, CABA, 1280, Argentina; b Cardiology Department, Hospital Británico de Buenos Aires, Perdriel 74, CABA, 1280, Argentina; c Esophageal and Gastric Surgery Department, Hospital Británico de Buenos Aires, Perdriel 74, CABA, 1280, Argentina

**Keywords:** Transesophageal echocardiography, Esophageal perforation, Minimally invasive, Endoscopic approach

## Abstract

**Introduction:**

Esophageal perforation is a rare and severe complication following transesophageal echocardiography (TEE) that carries high morbidity and mortality rates. Management of these perforations usually requires complex open surgeries. We present the case of an esophageal perforation following TEE treated with a combined approach of upper endoscopy and left cervicotomy.

**Presentation of case:**

An 80 y/o male patient underwent a diagnostic TEE for mitral regurgitation. After discharge patient consulted back on the same day to the Emergency Department and a perforation of the cervical esophagus was diagnosed associated to an air-fluid collection in the mediastinum. The patient was treated with endoscopic closure of the perforation and left cervicotomy for mediastinal drainage. Patient was discharged home on POD 31 after full recovery. A written consent was previously obtained, and Institutional Review Board approval was not needed.

**Discussion:**

Although not frequently seen, complications following TEE can be devastating if not diagnosed and treated early. Endoscopic closure of an esophageal perforation is a safe and feasible option with the already known advantages of a minimally invasive approach. Surgeons should have high suspicion if a patient present with characteristic symptoms after an uneventful procedure.

**Conclusion:**

Esophageal perforation is a very rare complication of TEE. High suspicion is mandatory to reach prompt diagnosis and install effective treatment. Primary closure of the perforation is the treatment of choice, and the endoscopic approach is a safe and feasible option in high volume centers.

## Highlights

• Esophageal perforation after ambulatory Transesophageal Echocardiography is rare.
• High suspicion in crucial to establish a diagnosis.
• If discovered early, treatment of esophageal perforation could have good outcome.
• Endoscopy plays an important role in the diagnosis and treatment.
• The endoscopic approach is a safe and feasible option to avoid major surgery.

## 1 Introduction

Transesophageal Echocardiography (TEE) plays an important role in the diagnosis, treatment, and surveillance of different cardiac pathologies [1]. Esophageal perforation (EP) is a severe but rare complication that may occur in patients undergoing TEE, with an incidence reported in large series below 0.1% [2,3]. Mortality rate reaches almost 30% and its associated to the risk of mediastinitis and sepsis [1,4]. Prompt diagnosis and effective treatment are crucial in the management of these patients that, usually, demand complex open surgeries for closure or diversion of the esophagus with drainage of the mediastinum. We present the case of a patient with an EP after a diagnostic TEE treated miniinviasively with an endoscopic/cervicotomy approach, achieving good outcomes for the patient. This case report has been reported in line with the SCARE checklist [5].

## 2 Presentation of case

**Fig. 1:**
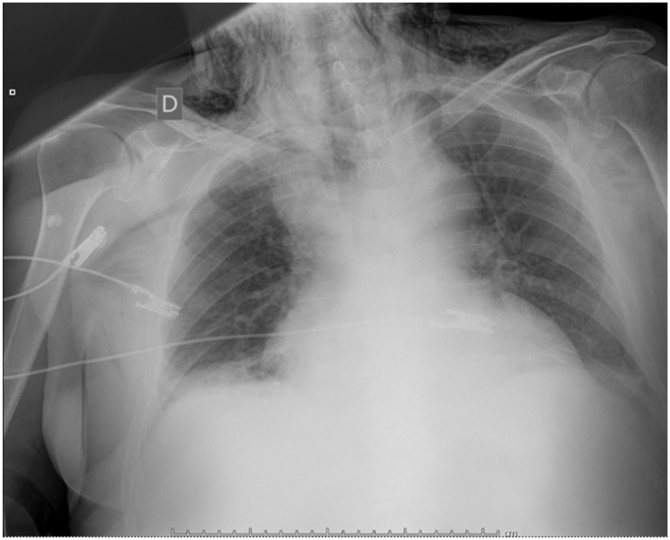
AP chest x-ray shows widened mediastinum.

An 80 years old male patient with a personal history of hypertension, hypercholesterolemia, hypothyroidism, atrial fibrillation and prostatic adenocarcinoma underwent a diagnostic TEE for assessment of a mitral regurgitation. The procedure was performed using local anesthesia with topical lidocaine. It was accomplished in an ambulatory setting, the passage of the probe was carried out without difficulty and resulted uneventful. 6 h after discharge, he consulted back to the emergency department complaining of fever and changes in his voice. Physical exam revealed axillar temperature up to 38 °C and subcutaneous emphysema in the neck and shoulders. Rest of the vital signs remained between normal ranges. White cell count at admission was within normal range and a chest x-ray showed widened mediastinum and subcutaneous emphysema (Fig. 1).

**Fig. 2:**
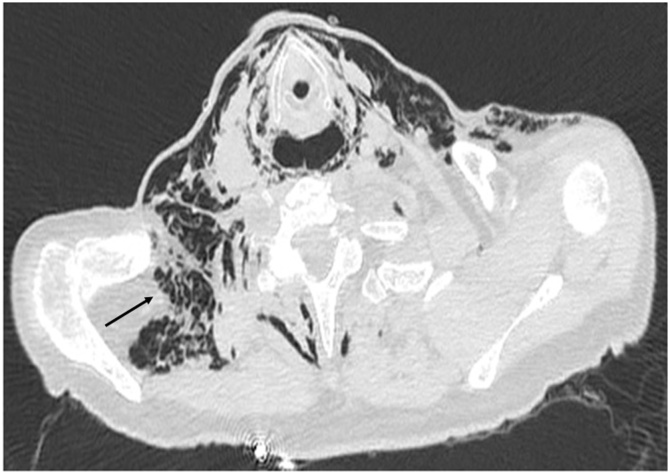
Chest CT shows subcutaneous emphysema.

**Fig. 3:**
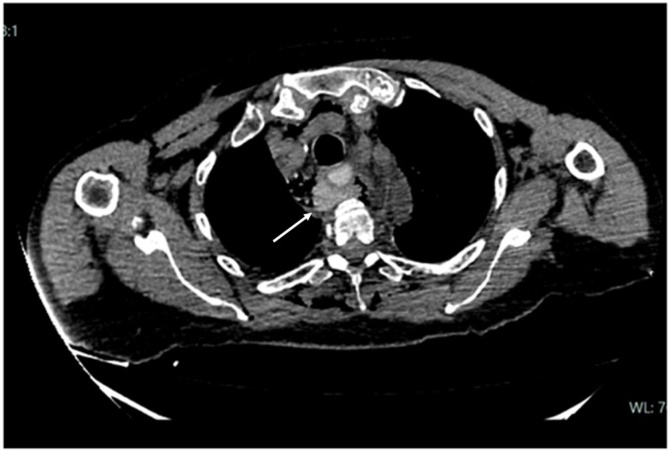
Mediastinal collection filled with oral contrast.

A double contrast Chest CT showed extensive subcutaneous emphysema, pneumomediastinum and an air-fluid collection filled with oral contrast in the posterior mediastinum (Fig. 2, Fig. 3).

**Fig. 4:**
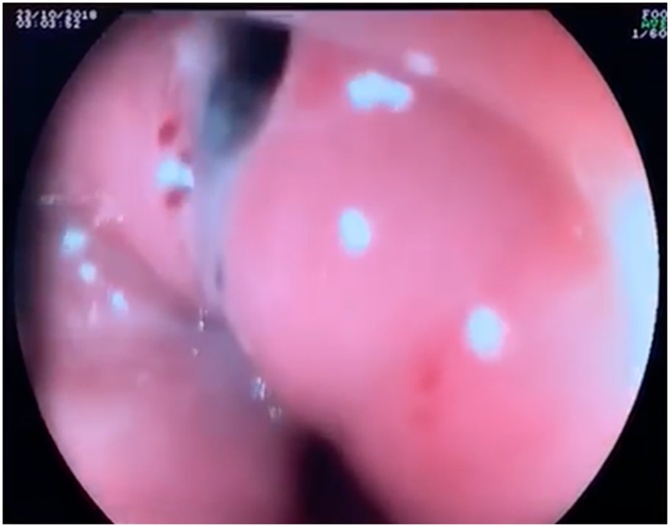
Upper endoscopy shows esophageal perforation of 10 mm.

With the suspicion of esophageal perforation and mediastinitis the patient was taken to the operating room for surgical intervention. We performed an upper endoscopy identifying a 10 mm esophageal perforation, at 15 cm from the incisors at the level of upper esophageal sphincter (Fig. 4) that was closed with four Resolution Clips (Boston Scientific). A nasogastric tube was placed for enteral nutrition. Secondly, the superior and posterior mediastinum was accessed through a left cervicotomy following the anterior border of the sternocleidomastoid muscle. A moderate amount of purulent fluid was drained and sent for cultures and 2 silicone drainage tubes were placed.

**Fig. 5:**
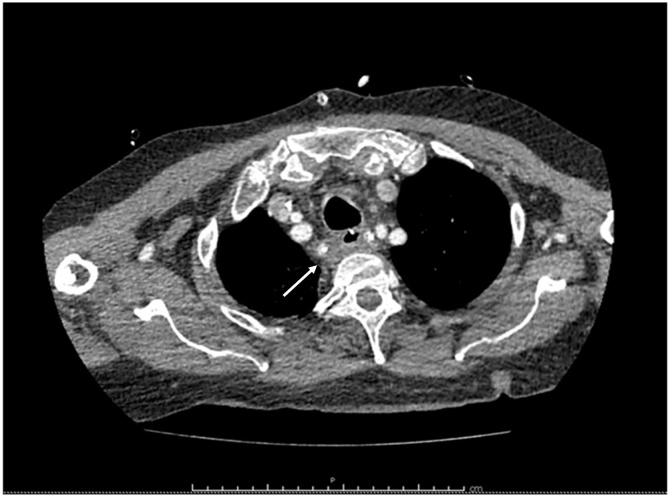
Cervical drainages next to a smaller collection.

After surgery the patient was transferred to the Intensive Care Unit. Antibiotic therapy (piperacillin/ tazobactam, vancomycin, fluconazole) and parenteral nutrition were administrated since POD 1 and enteral nutrition was initiated through the NG tube on POD 2. The patient had a favorable recovery being extubated on POD 5 and on POD 9 a CT scan revealed significant improvement of the subcutaneous emphysema and the air-fluid collections (Fig. 5).

On POD 24 amylase values from drainages were normal, and a barium swallow was done showing no contrast extravasation. Patient started an oral liquid diet with adequate tolerance and was discharged home on POD 31. Drainages were retired in clinic at POD 42. Medium term control revealed good patient recovery with no major complications.

## 3 Discussion

We present the case of a very rare complication of TEE that was managed successfully with a minimally invasive approach resulting in an “ad-integrum” patient recovery.

Transesophageal echocardiography (TEE) has been shown to be a safe procedure with a low morbidity rate that rounds 0.2% in large series [2]. Esophageal perforation (EP) is one of the most severe complications following TEE with a reported incidence between 0,03%–0,09% [5,6]. Although rare, these cases carry a high mortality rate, especially when associated to mediastinitis or sepsis. In a systematic review published in 2013, Sainathan et al. reported a mortality rate of 28%, with shock present at the time of detection of the EP in 40% of the deceased patients [1]. However, this mortality rate may be probably underestimated since the reported cases are usually the ones who survived and not those who deceased.

Several risk factors for EP following TEE have been described in previous publications [1]. Firstly, most of EP have been reported during intraoperative TEE, being far more common than in an ambulatory setting during a diagnostic procedure. In his review of 35 cases of EP, Sainathan reported that 75% occurred when TEE was performed in the operating room during cardiac surgery. Other case reports of TEE induced EP also occurred in an intraoperative setting [[7], [8], [9], [10], [11]]. The reason for this higher risk during intraoperative TEE may be explained by the prolonged, continuous pressure of the TEE probe to the esophageal mucosa, added to the possible low blood circulation in the esophagus during a cardiac surgery. Other known risk factors include chronic steroid therapy, prior chest radiation, severe cervical osteoarthritis, prior history of dysphagia, large left atrium causing esophageal distortion and hiatal hernia. Interestingly, our patient underwent a diagnostic, uneventful procedure in an ambulatory setting under conscious sedation, and he had no preoperative risk factors other than a hiatal hernia. Besides, the operator related no intraprocedural events such as resistance, multiple attempts, incomplete insertion or bloody secretions during the probe insertion.

We believe that a prompt diagnosis and initiating an effective treatment on time are crucial for the patient survival. A recent case report of an esophageal perforation in a patient who underwent cardiac surgery revealed that the diagnosis of perforation wasn’t confirmed until POD 7, with fatal outcomes for the patient, who ended up dying of cardiac arrest on POD 14 [10]. Sainathan et al. mentioned in their systematic review that the time of detection of an EP was negatively influenced by 3 factors: the presence of preprocedural risk factors, TEE performed in an intraoperative setting and a perforation in the thoracic esophagus. Fortunately, in our case, we could reach the diagnosis of esophageal perforation immediately after the patient consulted back to the ER on the first day after the TEE was performed. In concordance with Sainathans review, our patient didn’t have any risk factor other than a sliding hiatal hernia, the TEE wasn’t performed in an intraoperative setting and the perforation occurred in the cervical or upper thoracic esophagus. Probably, we could detect the perforation because the patient had unequivocal symptoms of esophageal perforation such us subcutaneous emphysema, voice changes and SIRS, allowing us to have a high clinical suspicion.

Primary repair is the modality of choice when dealing with esophageal perforations, especially when diagnosed during the first 24 h. Historically, these were treated with complex surgeries such as esophageal resection or diversion requiring large thoracotomies. In the last decades, endoscopy raised not only as a diagnostic tool but also as a treatment choice for EP. Multiple treatment options appeared as the result of the evolving techniques, such as hemostatic clips, larger over the scope clips (OTSC), a variety of self-expandable esophageal stents and endoscopic vacuum assisted closure or Endo Sponge. Despite this, the endoscopic approach is rarely used, with only 6% of reported cases treated in this way. Herbold et al. reported their single center series of six cases of TEE induced esophageal perforation, none of them treated with open surgery [12]. Two perforations were located in the cervical esophagus and managed with conservative treatment while 4 cases had a thoracic esophageal perforation and were treated endoscopically: three with self-expandable stents and one case with stent and endoscopic vacuum therapy. Similar to Herbold et al., we opted for an endoscopic primary closure of the perforation with four Resolution Clips but we had to drain the mediastinal collections through a surgical approach. Thus, we chose a left cervical oblique incision following the anterior edge of the sternocleidomastoid muscle. This allowed us to access the superior and posterior mediastinum in order to treat the mediastinitis. We believe that this minimally invasive approach, avoiding a thoracotomy, allowed the patient to have an ad integrum recovery with no complications or adverse events.

In conclusion, esophageal perforation is a very rare complication of TEE. High suspicion is mandatory in order to reach a prompt diagnosis and install an effective treatment on time. Primary closure of the perforation is the treatment of choice, and the endoscopic approach is a safe and feasible option in high volume centers.

## Sources of funding

There were no sources of funding.

## Ethical approval

Ethical approval has been exempted by our institution.

## Consent

Written informed consent was obtained from the patient for publication of this case report and its accompanying images.

## Author’s contribution

Facundo Iriarte: Investigation; data collection; methodology; writing - original draft; visualization. German A. Riquelme: data collection; writing - original draft; visualization. Pablo Sorensen: Resources, validation. Daniel E. Pirchi: Validation; project administration. Matias Mihura Irribarra: writing - review & editing; resources; supervision; validation.

## Registration of research studies

N/A.

## Guarantor

Matias Mihura Irribarra accepts full responsibility for the article.

## Provenance and peer review

Not commissioned, externally peer-reviewed.

## Declaration of Competing Interest

Facundo Iriarte, German A. Riquelme, Pablo Sorensen, Daniel E. Pirchi and Matias Mihura Irribarra declare that there is no conflict of interest regarding the publication of this article.
